# Targeting the transcriptional activity of STAT3 by a novel fusion protein

**DOI:** 10.1186/s12885-022-09837-1

**Published:** 2022-07-10

**Authors:** Yanqiong Chen, Wenting Zhang, Xiufeng Bai, Yi Liu

**Affiliations:** 1 National Clinical Research Center for Geriatrics and Laboratory of Human Disease and Immunotherapies, West China Hospital, Sichuan University, Sichuan Province, Chengdu, 610041 China; 2 Research Institute of Inflammation and Immunology (RIII), Frontiers Science Center for Disease-related Molecular Network, West China Hospital, Sichuan University, Chengdu, China; 3 Rare Diseases Center, West China Hospital, Sichuan University, Chengdu, 610041 China

**Keywords:** KRAB, STAT3, Transcription, cancer, Autoimmune diseases, Neurodegenerative diseases, Male infertility

## Abstract

**Background:**

The continuous activation of transcription factors drives many diseases, including tumors, autoimmune disease, neurodegenerative disease, and male infertility. Thus, Blocking the transcriptional activity of these proteins may inhibit disease progression. In this study, we developed a new method to specifically inhibit the activity of the transcription factor STAT3.

**Methods:**

Fusing the transcriptional inhibitory domain KRAB with STAT3 successfully blocked the transcription activity of STAT3 in cancer cells without affecting its function in the mitochondria and lysosomes.

**Results:**

the expression of KRAB-STAT3 fusion protein inhibited the growth of tumor cells.

**Conclusions:**

The KRAB-STAT3 fusion protein provides a novel approach for drug development for the treatment of cancer or autoimmune diseases.

**Supplementary Information:**

The online version contains supplementary material available at 10.1186/s12885-022-09837-1.

## Background

The abnormal activation of numerous signaling pathways lead to diseases such as tumors, autoimmune diseases, neurodegenerative diseases, and male infertility. Attempts have been made to block the continuous transcriptional activation, such as inducing protein degradation through (Proteolysis-targeting chimeras, PROTAC ) [1], blocking protein-protein interaction through antibodies [2], and silencing or knocking out gene expression using (RNA interference, RNAi ) [3] or Clustered regularly interspaced palindromic repeats (CRISPR)- [4]. The transcription factor signal transducer and activator of transcription 3 (STAT3) is a critical promoter during tumorigenesis [5]. In the inactive state, STAT3 is a monomer and localized in the cytoplasm. When phosphorylated on tyrosine 705 (Y705), STAT3 forms a homodimer with another STAT3 or a heterodimer with other STAT family members. Then, STAT3 is transpositioned into the nucleus by importin-β1 [6] or importin-a3 [7]. The nucleus STAT3 binds to the promoter of the target genes, including *c-Myc*, *cyclinD1*, and *MCL-1* [8, 9], and initiates transcription.

If STAT3 is ov2ctivatedated, it may cause cancer or autoimmune diseases. Many small molecules targeting the phosphorylation site of STAT3 have been developed [10–12]. However, due to the structural similarity between STAT proteins and the existence of a heterodimer, it is difficult to specifically inhibit the transcription activity of STAT3 by small molecules. STAT3 also plays a critical role in mitochondria [13] and lysosomes [14]. Moreover, STAT3 diminished markedly in the cell by PROTAC, RNAi, or CRISPR may cause side effects. Therefore, developing a novel strategy to block the abnormal STAT3 activity is an urgent requirement.

Kruppel-associated box (KRAB) is a transcriptional repressor domain commonly found in eukaryotic zinc finger proteins [15]. It is fused with Cas9 to silence the gene expression by inducing the transcriptional inhibition [16, 17]. In the present study, we fused the KRAB domain to the N terminal of STAT3 to make a Trojan horse and found that this KRAB-STAT3 (K-S) fusion protein specifically inhibits STAT3 transcription and does not affect the function of the mitochondria and lysosomes.

## Materials and methods

### Mice

Six–eight-week-old nude mice were purchased from Charles River (Beijing, China) and maintained at the Laboratory Animal Center of Sichuan University. An equivalent of 1 × 10^5^ cells was injected subcutaneously, and tumor volume was measured with a caliper (length and width). At the end of the experiment, all animals were anesthetized with 10% pentobarbital sodium and killed by cervical dislocation. The present study was approved by the Animal Ethics Committee of Sichuan University (20211478A). All the experimental protocols were approved and carried out in accordance with the relevant guidelines and regulations of the Animal Ethics Committee of Sichuan University and (Animal Research: Reporting of In Vivo Experiments, ARRIVE) guidelines.

### Cell lines and reagents

Cell lines A375 and HeLa were purchased from (American Type Culture Collection, ATCC) and grown at 37 °C in the presence of 5% CO_2_ in Dulbecco’s modified Eagle’s medium (DMEM, Biological Industries 01-052-1A) with high glucose and supplemented with 10% fetal bovine serum (FBS, Biological Industries 04-007-1A), 2 mmol/L L-glutamine, and antibiotic-antimycotic solution (1X, Gibco 15240112). The stable clones of A375 and HeLa KRAB-linker-STAT3a-Flag were selected under 6 μg/mL puromycin for 7 days.

### Plasmid construction and lentivirus production

The pLVX-IRES-Puro plasmid was purchased from Clontech Laboratories (632183). KRAB-Linker-STAT3a-3XFlag coding sequence was synthesized by Beijing TsingKe Biotechnology and cloned into pLVX-IRES-Puro between *EcoRI* and *BamHI* restriction endonucleases. The amino acid sequence of KRAB-Linker-STAT3a was uploaded to Addgene (185733). psPAX2 and pMD2.G was gifts from Didier Trono (Addgene plasmid # 12260; http://n2t.net/addgene:12260; RRID: Addgene_12,260 and Addgene plasmid # 12259; http://n2t.net/addgene:12259; RRID: Addgene_12,259, respectively). HEK293T cells were transfected with Plvx-KRAB-Linker-STAT3a-3XFlag plasmid, psPAX2 plasmid, and pMD2.G plasmid in DMEM using polyethylenimine (PEI). At 48- and 72-h post-transfection, the cell culture media was collected and stored at − 80 °C.

### Cell proliferation analysis in vitro

Cell proliferation was monitored using BeyoClick™ EdU-555 (Beyotime Biotechnology, C0075L) according to the manufacturer’s instructions. Briefly, 1 × 105 cells were cultured in six-well plates with 10 mM EdU containing complete medium for 2 h and fixed with Immunol Staining Fix Solution (Beyotime Biotechnology, P0098) at room temperature for 15 minutes. Then, the cells were permeabilized with Immunostaining Permeabilization Buffer with Triton X-100 (Beyotime Biotechnology, C0075L) at room temperature for 15 minutes. Subsequently, 500 μL Click Additive Solution (Click Reaction Buffer: 430 μL, CuSO_4_: 20 μL, azide 555: 1 μL, and Click Additive Solution 50 μL) and Hoechst 33342 were added to the cell and incubated in the dark at room temperature for 30 minutes. Finally, cell proliferation was analyzed using FACSAria SORP (BD Biosciences, USA) and FlowJo program.

### Immunofluorescence

Cells grown on coverslips were fixed with Immunol Staining Fix Solution at room temperature for 5 minutes and blocked in QuickBlock™ Blocking Buffer for Immunol Staining (Beyotime P0260) at 37 °C for 30 minutes. Then, the cells were washed with phosphate-buffered saline (PBS, pH 7.2) for 30 min and incubated with primary antibodies at a 1:100 dilution in 5% bovine serum albumin (BSA) at 4 °C overnight, followed by incubation with secondary antibodies at a 1:200 dilution at 37 °C for 1 h and Hoechst 33342 staining at room temperature for 5 minutes. Finally, the cells were mounted with Antifade Mounting Medium (Beyotime Biotechnology, P0128) and examined under a fluorescence microscope (Olympus, IX73, Japan). The primary antibodies used in this study were mouse anti-Stat3 monoclonal antibody (Cell Signaling Technology, 9139, USA), rabbit anti-Flag monoclonal antibody (Cell Signaling Technology, 14,793), and rabbit anti-c-Myc monoclonal antibody (Cell Signaling Technology, 18,583) and the secondary antibodies were donkey anti-rabbit IgG (H + L) Alexa Fluor Plus 488 (Invitrogen, A32766, USA) and donkey anti-mouse IgG Alexa Fluor Plus 555 (Invitrogen, A32773).

### Western blotting

The cells were lysed in cell lysis buffer containing 1 mM protease inhibitor phenylmethanesulfonyl fluoride (PMSF) (MedChemExpress, HY-B0496) for immunoprecipitation (Beyotime Biotechnology, P0013) and Western blot. Mouse anti-Stat3 monoclonal antibody (Cell Signaling Technology, 9139), rabbit anti-Phospho-Stat3 (Tyr705) monoclonal antibody (Cell Signaling Technology, 9145), rabbit anti-c-Myc monoclonal antibody (Cell Signaling Technology, 18,583), rabbit anti-Cyclin D1 monoclonal antibody (Cell Signaling Technology, 55,506), rabbit anti-MCL1 monoclonal antibody (Huabio, ET1606-14), and mouse anti-GAPDH monoclonal antibody (Huabio, EM1101) were used at a 1:1000 dilution in 5% BSA at 4 °C overnight. The secondary antibodies were horseradish peroxidase (HRP)-linked anti-mouse IgG (Cell Signaling Technology, 7076S) and HRP-linked anti-rabbit IgG (Cell Signaling Technology, 7074S) used at a 1:5000 dilution at 37 °C for 1 h. The immunoreactive bands on the (Polyvinylidene Fluoride, PVDF) membranes were evaluated using a chemiluminescence instrument and quantified using Image J software (NIH).

### Transwell assay

For the migration assay, 1 × 10^5^ cells were cultured in the upper chamber with 200 μL FBS-free DMEM medium, while 600 μL DMEM medium with 10% FBS was added to the lower chamber. Cells were incubated at 37 °C in a 5% CO_2_ for 48 h. Subsequently, the cells on the top side of the upper chamber were removed gently using a wet cotton swab, and the remaining cells on the bottom side were stained with 0.1% crystal violet and analyzed under a microscope (Olympus, CKX41).

### Quantitative real-time PCR analysis

RNA was extracted using TRIzol (Invitrogen, 10,296,010), and cDNA was synthesized using TransScript II One-Step RT-PCR SuperMix (TransGen Biotech, AH411-02, China). PerfectStart Green qPCR SuperMix (TransGen Biotech, AQ601-01) was used to prepare the PCR reaction mixture containing 1 μg of cDNA. *Gapdh* was used as a reference gene. The PCR amplification was conducted on the CFX96 thermal cycler (Bio-Rad). The primer sequences were downloaded from Primer-bank: *Stat3* (ID: 47080104c1), *c-Myc* (ID: 239582723c1), *cyclinD1* (ID: 77628152c1), *Mcl1* (ID: 11386165a1), and *Gapdh* (ID: 378404907c1).

### Lysosomal pH estimation by flow cytometry

Lysosomal pH was measured using the lysosomal pH detection kit (Beijing Biolab Technology, HR8268) according to the manufacturer’s instructions. Briefly, 1 × 10^5^ A375 cells were cultured in the cell culture medium containing P02 for 5 min. Then, lysosomal pH was analyzed on FACSAria SORP (BD Biosciences) using FlowJo software.

### Flow cytometric sorting

Flow cytometric sorting was performed as described previously [18]. A375 and HeLa cells were infected with Plvx-KRAB-STAT3-3XFlag lentivirus for 7 days. The cells were digested with trypsin (0.25 mg/mL, Gibco 25,300,054), and the supernatant was removed by centrifugation (500×*g*, room temperature, 3 minutes). The cells were filtered through a 70-μm membrane, sorted with FACSAria SORP (BD Biosciences), and analyzed with FlowJo software.

### Examination of cellular ATP content

The ATP level was detected using ATP Assay Kit (Beyotime Biotechnology, S0026), according to the manufacturer’s instructions. Briefly, the cells were lysed using 200 μL lysis buffer and clarified by centrifugation at 4 °C, 12000×*g* for 5 minutes. Then, 200 μL working solution was added to 20 μL supernatant, and the luminescence was measured on a microplate reader (Molecular Devices, SpectraMax i3x).

### Cell component separation

Mitochondria were isolated using the Cell Mitochondria Isolation Kit (Beyotime, C3601), according to the manufacturer’s instructions. Briefly, cells were lysed using lysis buffer (250 mM sucrose; 10 mM Tris-HCl (pH 7.4), and 1 mM EDTA) on ice for 10 minutes, and the cell debris was removed by centrifugation at 4 °C, 1000×*g* for 5 min. The supernatant was clarified by centrifugation at 12000×g for 30 minutes; the mitochondria were in the sediment, and the cytoplasmic component was in the supernatant. Subsequently, the nuclear protein was separated using the Nuclear and Cytoplasmic Protein Extraction Kit (Beyotime, P0027). Briefly, cells were lysed using Buffer A, vortexed for 5 s, and placed on ice for 15 minutes. Then, Buffer B was added, and the mixture was vortexed for 5 s and incubated on ice for 1 minute, followed by centrifugation at 4 °C, 16000×*g* for 5 minutes. The cytoplasmic component was in the supernatant, and nuclear proteins were in the precipitate.

### Statistical analysis

Each set of experiments was repeated at least three times. All statistical analyses were performed using GraphPad Prism 9. The quantitative data were presented as mean ± standard deviation (SD).

## Results

### Construction of the KRAB-STAT3 fusion protein

**Fig. 1:**
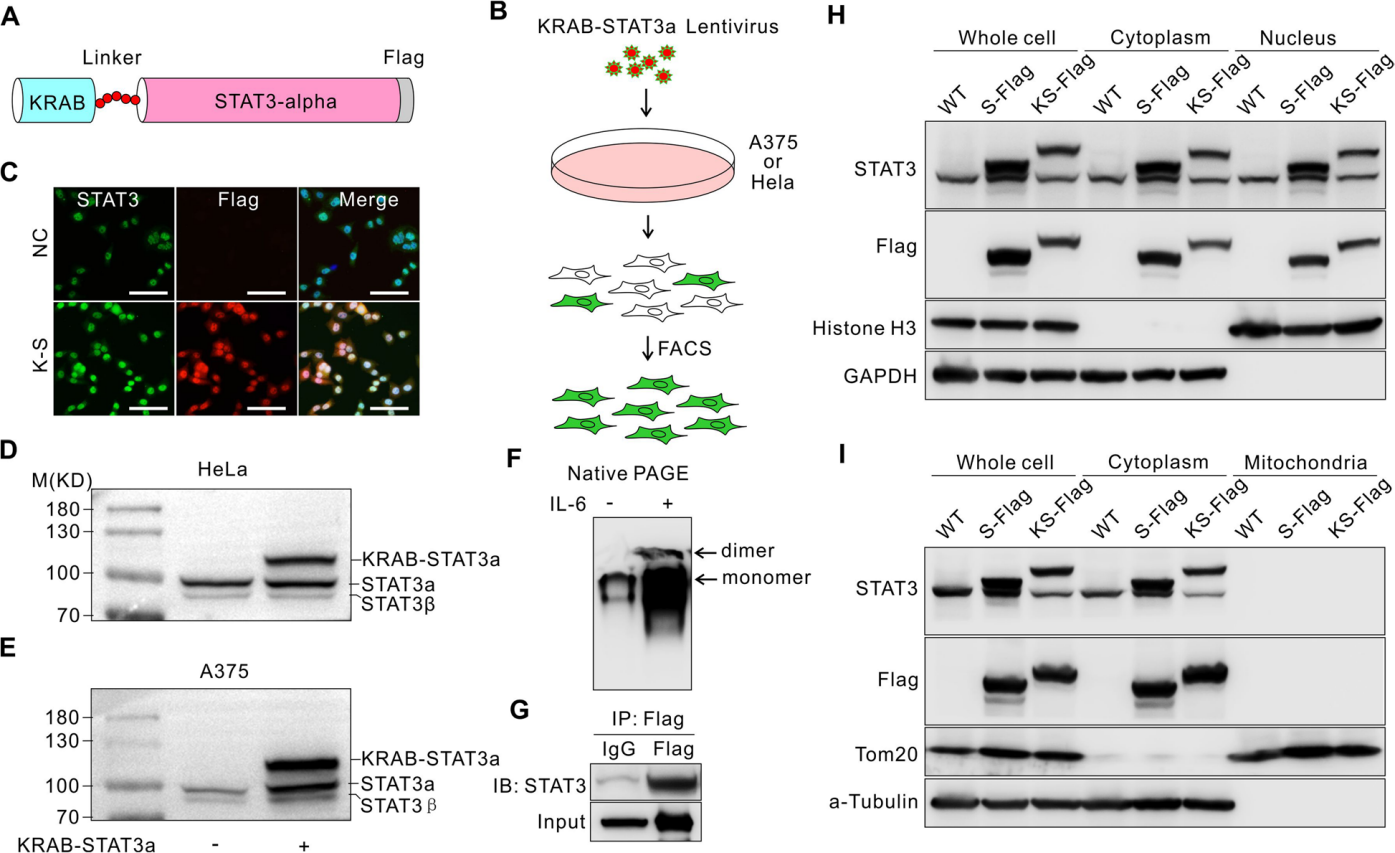
Construction of the KRAB-STAT3 fusion protein. **A**, Model of KRAB-STAT3 secondary structure. **B**, Graphic model shows the workflow of the monoclonal cell line screen. **C**, Immunofluorescence of the expression of STAT3 and Flag in monoclonal Hela cells. K-S: KRAB-STAT3. Bars = 50 um. **D, E**, Western blot of the expression of KRAB-STAT3 fusion protein in monoclonal HeLa and A375 cells. **F,** Western blot of Native PAGE of KRAB-STAT3-3xFlag using anti-Flag antibody. **G**, Co-immunoprecipitation of KRAB-STAT3-3xFlag fusion protein using anti Flag antibody, and endogenous STAT3 was detected using anti-STAT3 antibody at around 86 kDa. **H**, **I**, STAT3 and KRAB-STAT3-3xFlag was detected in the cytoplasm, mitochondria, and nucleus using Western blot. Original blots were in Supplementary Fig. 1

STAT3 has four isoforms (α, β, γ, and δ) [19, 20]. Alternative splicing generates STAT3α and STAT3β; STAT3α is the full-length form of STAT3 and acts as a tumor promoter, while STAT3β lacks a transcriptional activation domain. Therefore, STAT3α was used in this study. Moreover, STAT3 was activated to form a homodimer or heterodimer to bind to DNA. Owing to its proximity to the DNA binding domain, the C-terminal of STAT3 is not suitable for modification. Therefore, we fused the KRAB domain to the N-terminal of STAT3 and the flag sequence to the C-terminal. Also, a linker was inserted between KRAB and STAT3. The total length of KRAB-Linker-STAT3-Flag was 868 amino acids, and the total molecular weight was 111 kDa (Fig. 1A). Then, we packaged the virus containing the KRAB-STAT3 coding sequences in 293 T cells to obtain lentiviral particles and infected the A375 or HeLa cells. Subsequently, the cells were screened under puromycin selection, and the KRAB-STAT3-expressing cells were obtained by flow cytometry (Fig. 1B). To further confirm the KRAB-STAT3 fusion protein-expressing cell line, we detected the Flag tag using immunofluorescence and identified the cells positive for KRAB-STAT3 fusion protein by flag tag (Fig. 1C). In addition, Western blot analysis of STAT3 showed that STAT3α was the main form of endogenous STAT3. Also, a 110-kDa fusion protein was detected above the band of STAT3α, which was KRAB-STAT3 fusion protein. The results further confirmed the expression of KRAB-STAT3 fusion protein in monoclonal cell lines (Fig. 1D, E). KRAB-STAT3 forms dimers in HEK293 cells after interleukin (IL)-6 treatment (Fig. 1F). The fusion protein also interacts with endogenous STAT3 (Fig. 1G). Then, the cellular components were isolated, and KRAB-STAT3 was located in the cytoplasm and nucleus (Fig. 1H) but not in mitochondria (Fig. 1I).

### KRAB-STAT3 inhibits *STAT3* target gene expression

**Fig. 2:**
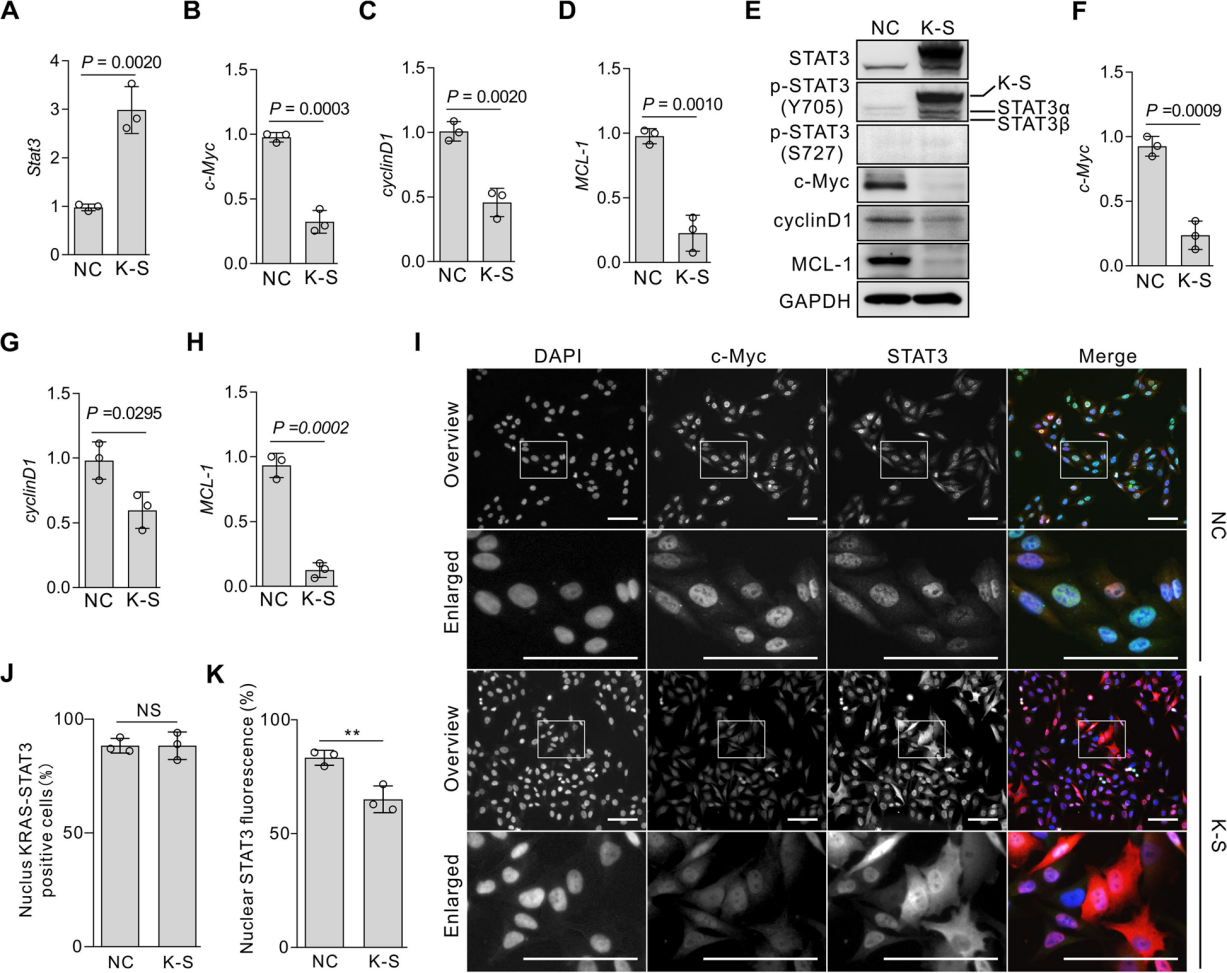
KRAB-STAT3 inhibits the endogenous STAT3 transcriptional activity. **A–D**, PCR of STAT3 and target genes in the control and monoclonal A375 cells represented as mean ± SD; *n* = 3; Student’s t-test. **E–H**, Western blot of STAT3 and target genes in monoclonal A375 cells represented as mean ± SD; *n =* 3; Student’s t test, original blots were in Supplementary Fig. 2. **I-J,** Immunofluorescence of c-Myc and STAT3 in monoclonal A375 cells, NS: no significance. Arrows show that the cytoplasm retained STAT3. Bars = 50 μm

STAT3 is activated in tumor cells, resulting in overexpression of the downstream target genes. To determine whether KRAB-STAT3 negatively regulates the expression of downstream target genes, we used RT-PCR to detect the mRNA level of the target genes. Also, the expression of KRAB-STAT3 reduced the transcription of *c-Myc*, *CyclinD1*, and *Mcl-1* (Fig. 2A–D). However, the decrease in the mRNA level was not equal to the decrease in protein level because many events, such as mRNA longevity and ribosome translation efficiency, could cause inconsistency between the mRNA level and protein level [21]. Therefore, we detected the protein level of these target genes by Western blot and found that the levels of c-Myc, CyclinD1, and Mcl-1 proteins were decreased significantly (Fig. 2E–H). In addition, we used immunofluorescence and found that the expression of c-Myc was decreased in KRAB-STAT3-expressing cells. We also found maximal STAT3 accumulation in the nucleus in most KRAB-STAT3-expressing cells (Fig. 2I, J). However, the percentage of nuclear STAT3 fluorescence [22] is less in KRAB-STAT3-expressing cells (Fig. 2K).

### KRAB-STAT3 promotes apoptosis and inhibits proliferation

**Fig. 3:**
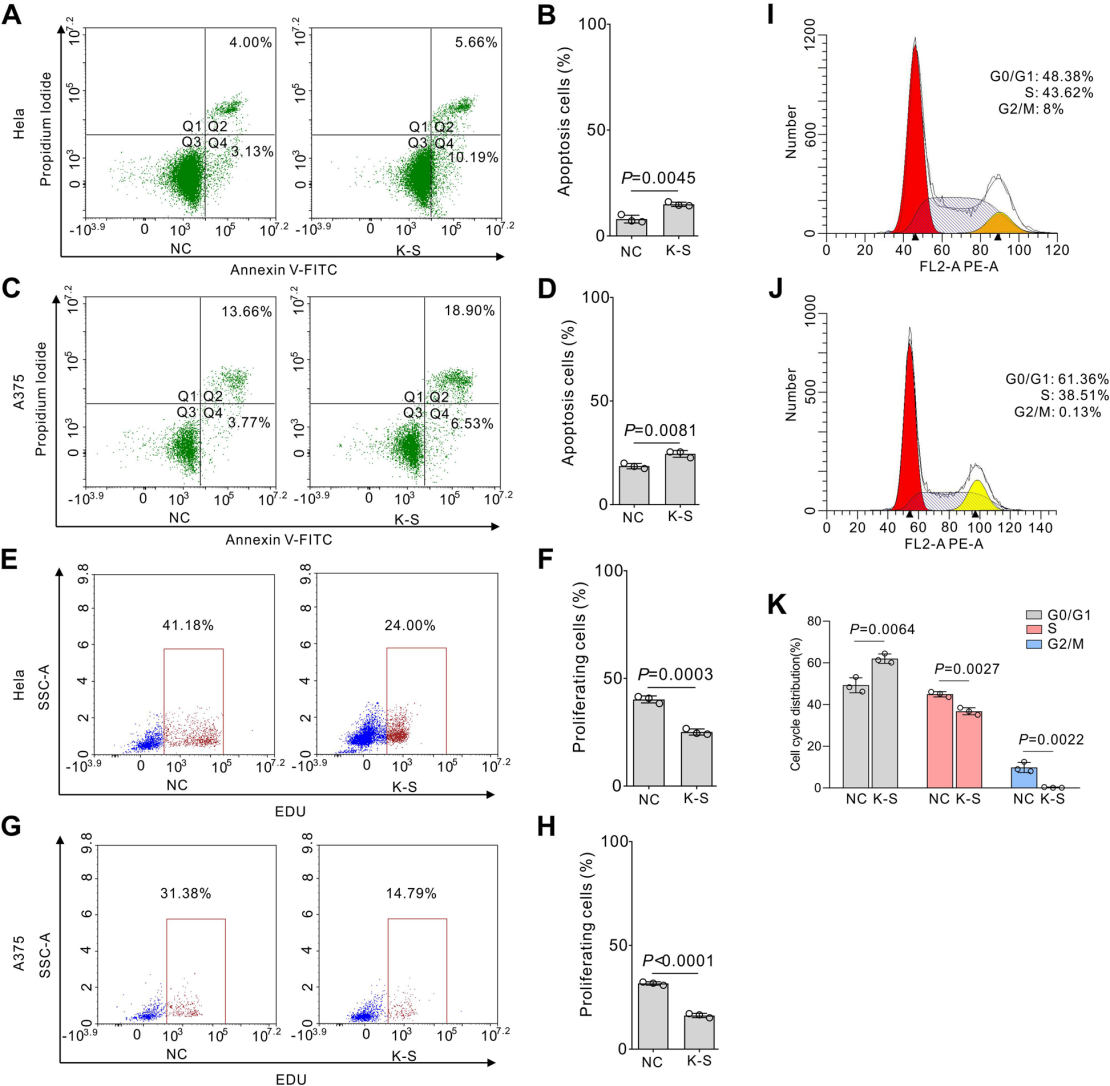
KRAB-STAT3 promotes apoptosis and inhibits proliferation. Flow cytometry analysis of apoptosis in HeLa (**A**, **B**) and A375 (**C**, **D**) cells represented as mean ± SD; *n =* 3; Student’s t-test. Flow cytometry analysis of cell proliferation in HeLa (**E**, **F**) and A375 (**G**, **H**) cells represented as mean ± SD; *n =* 3; Student’s t-test. **I–K**, Cell cycle analysis of A375 cells represented as mean ± SD; *n =* 3; Student’s t-test

STAT3 is a widely recognized oncogene, and the continuous activation of STAT3 causes malignant transformation of normal cells [23]. *MCL1*, the target gene of STAT3, inhibits apoptosis [24]. The inhibition of phosphorylation of STAT3 induces apoptosis in cultured pancreatic cancer cells [25] and solid and hematological tumors [26]. To further confirm the role of KRAB-STAT3 on apoptosis, we detected the cell death rate in the fusion protein-transfected HeLa and A375 cells by flow cytometry. Also, the expression of KRAB-STAT3 slightly increases the apoptosis of HeLa (Fig. 3A,B) and A375 cells (Fig. 3C,D). Moreover, the proliferation-promoting protein cyclinD1 is the target gene of STAT3 [27]. STAT3 promotes cell growth in neurocytes [28], hepatocellular carcinoma cells [29], and colon cancer cells [30]. Next, the overexpression of KRAB-STAT3 decreased the cell proliferation in HeLa (Fig. 3E,F) and A375 cells (Fig. 3G,H). Cell cycle analysis showed that KRAB-STAT3-expressing arrested the A375 cells in G0 and G1 phases (Fig. 3I–K).

### KRAB-STAT3 inhibits the migration and invasion in vitro

**Fig. 4:**
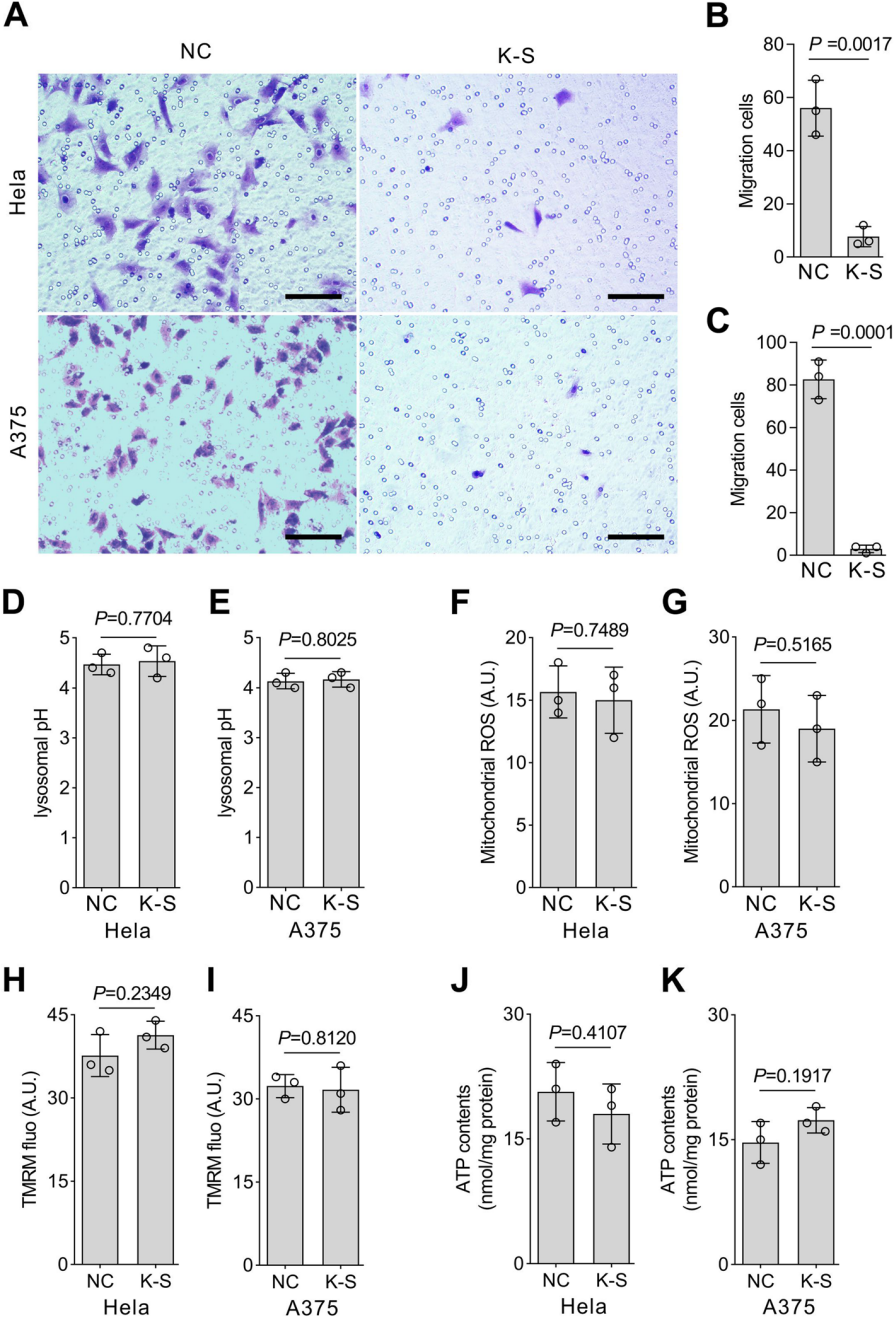
KRAB-STAT3 inhibits cell migration and invasion. **A**, Transwell assay of control and KRAB-STAT3-expressing HeLa and A375 cells. K-S: KRAB-STAT3. Bars = 50 μm. **B**, Migrated cells per view of control and HeLa cells. **C**, Migrated cells per view of control and A375 cells represented as mean ± SD; *n =* 3; Student’s t-test. **D**, **E**, Lysosome pH analysis of HeLa and A375 cells. **F-G**, Mitochondria ROS production detection using mitoSOX, A.U., Any unit. **H-I**, Mitochondrial membrane potential detection using TFRM. **J-K**, ATP content in HeLa and A375 cells represented as mean ± SD; *n =* 3; Student’s t-test

Invasion and metastasis are major characteristics of tumor cells and also the leading causes of cancer-related deaths [31]. The over-activation of STAT3 promotes cell invasion and metastasis [32, 33]. STAT3 inhibitors, piperine [11], pyrimethamine [10], and salidroside [34], inhibit cancer cell metastasis. Transwell assay demonstrated that KRAB-STAT3 expression reduced the invasiveness of HeLa and A375 cells (Fig. 4A–C). Moreover, lysosomal pH, mitochondria reactive oxygen species (ROS), mitochondrial membrane potential, and ATP production were detected using flow cytometry, and no difference was observed between KRAB-STAT3 fusion protein-expressing cells and control cells (Fig. 4D–K).

### KRAB-STAT3 inhibits tumor growth in vivo

**Fig. 5:**
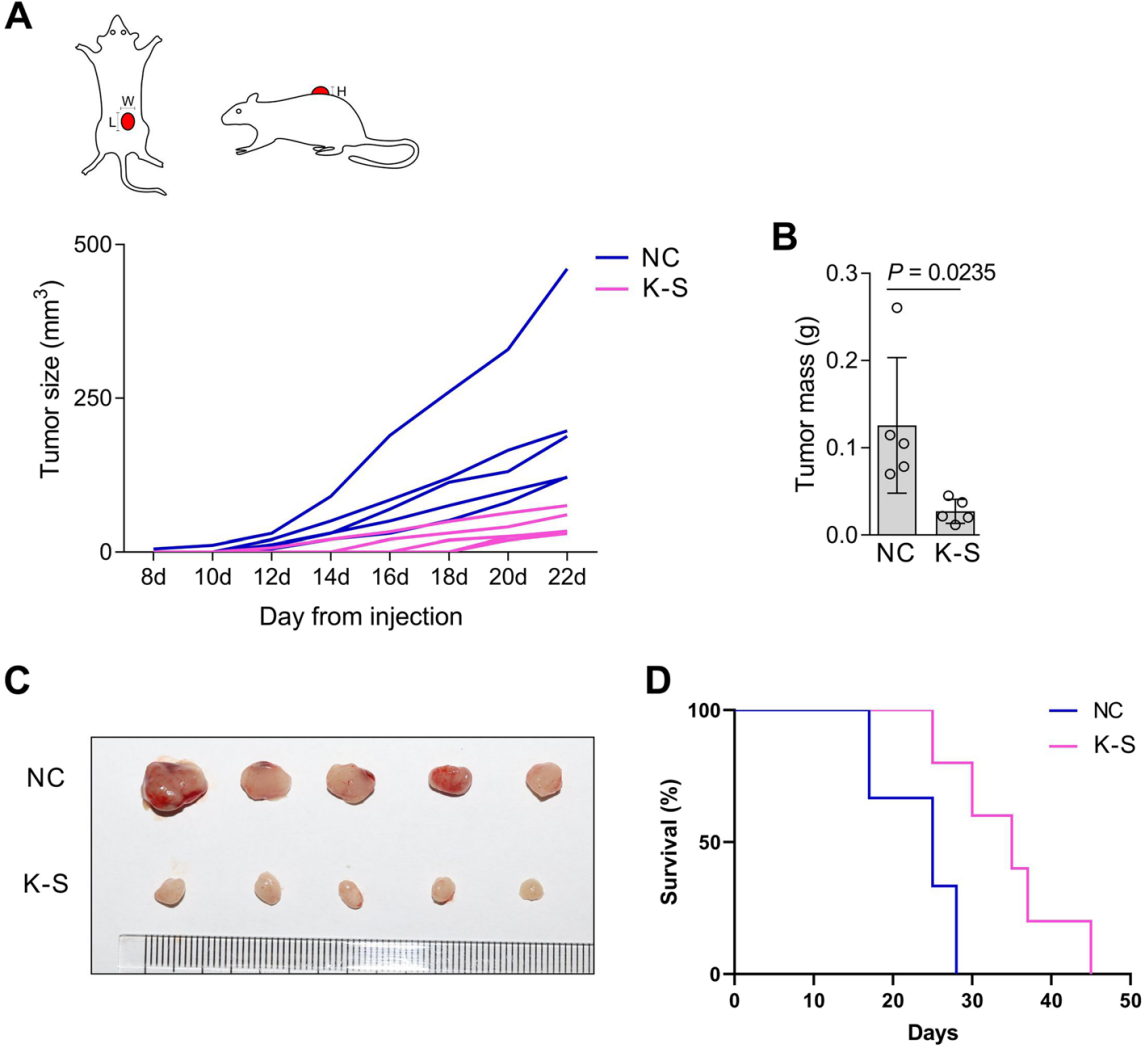
KRAB-STAT3 inhibits cell growth in vivo. **A**, Model of tumor measurement (W: width, L: long, H: high) and tumor volume on nude mice from day 8 post-subcutaneous injection of A375 cells. **B**, Tumor mass on day 22 post tumor cell injection represented as mean ± SD; *n =* 3; Student’s t-test. K-S: KRAB-STAT3. **C**, Size of the tumor on day 22 post-tumor cell injection. K-S: KRAB-STAT3. **D**, Survival of nude mice after the injection of A375 cells via the caudal vein

To further elucidate the inhibitory effect of KRAB-STAT3 on the in vivo growth of cancer cells, the KRAB-STAT3-expressing A375 cells were implanted subcutaneously in nude mice. Next, we found that the expression of KRAB-STAT3 slows tumor growth (Fig. 5A). In addition, tumor weight and volume were lower in KRAB-STAT3-positive cells (Fig. 5B,C). To analyze the effect of fusion protein on the metastatic ability of A375 cells, A375 cells were implanted into nude mice by tail vein injection. We observed that the mice xenografted with KRAB-STAT3-expressing tumor cells had a prolonged survival time (Fig. 5D).

## Discussion

After dimerization, STAT3 formed a pincer-like structure. The head of the pincer was the DNA-binding and SH2 domains, located in the middle and C-terminal of STAT3. If the KRAB domain was fused to the C-terminal of STAT3, the protein might not bind to DNA due to the severe steric hindrance. Therefore, KRAB domain was fused to the N-terminal of STAT3 in this study. Moreover, a linker was inserted between the KRAB domain and STAT3 to weaken the protein rigidity and maintain the transcriptional inhibitory activity of KRAB. Furthermore, STAT3 has two common isoforms: STAT3α and STAT3β. STAT3α but not STAT3β contains the transcriptional activation domain. Some studies suggested that the β-form STAT3 inhibits the transcription function of STAT3α competitively because it binds to the same DNA sequence. Moreover, STAT3α is widely known as an oncogene [19, 35]. Therefore, in this study, STAT3α was used to modulate the transcriptional function of endogenous STAT3. The percentage of nuclear STAT3 fluorescence was decreased in KRAB-STAT3 expressing cells, this may be caused by the disruption of the positive feedback loop of STAT3 signaling. *IL6* is known as a target gene of STAT3 [9, 36–38], which further act as an upstream activator of STAT3. KRAB-STAT3 reduces the transcription of *IL6* and thus the content of STAT3 in the nucleus. Further studies are needed to confirm this hypothesis.

Furthermore, STAT3 is localized in the mitochondria [39]. However, KRAB-STAT3 does not translocate into the mitochondria, which could be attributed to the low level of phosphorylation on serine 727 of STAT3. The expression KRAB-STAT3 inhibits cancer cell proliferation both in vitro and in vivo, caused by inhibition of STAT3 signaling through KRAB-STAT3. The over-activated STAT3 signaling in cancer cells induces target gene expression; these genes include *cyclinD1* (promotes cell growth) and the BCL2 family member *MCL-1* (reduces apoptosis). Taken together, the persistent transcript of these genes fuels tumor growth. However, when these are blocked by KRAB-STAT3, the tumor growth was slowed down. Therefore, KRAB-STAT3 provided a novel method to block tumor growth.

AAV is a widely used gene delivery vector [40]. Using AAV to deliver the target genes to tumor cells is a critical application. KRAB-STAT3 is only 2.6 kbp and can be packaged into AAV and delivered to cancer cells using tumor-targeting nanoparticles. Therefore, the fusion of the inhibitory domain to a transcript factor is a promising tool in disease treatment.

## Conclusion

The present study provides a new approach to treating cancer, which could be used in drug development to treat the diseases caused by out-of-control transcription factors.

## Supplementary Information

**Additional file 1.**

**Additional file 2.**

## Data Availability

The datasets generated and/or analysed during the current study are available in the addgene repository (ID: 186228) (https://www.addgene.org/186228/).
