# Supplementary figures and images for: Targeting the transcriptional activity of STAT3 by a novel fusion protein
Source: BMC Cancer. 2022 Jul 10;22:751. doi: 10.1186/s12885-022-09837-1 (PMC9271252; doi:10.1186/s12885-022-09837-1)

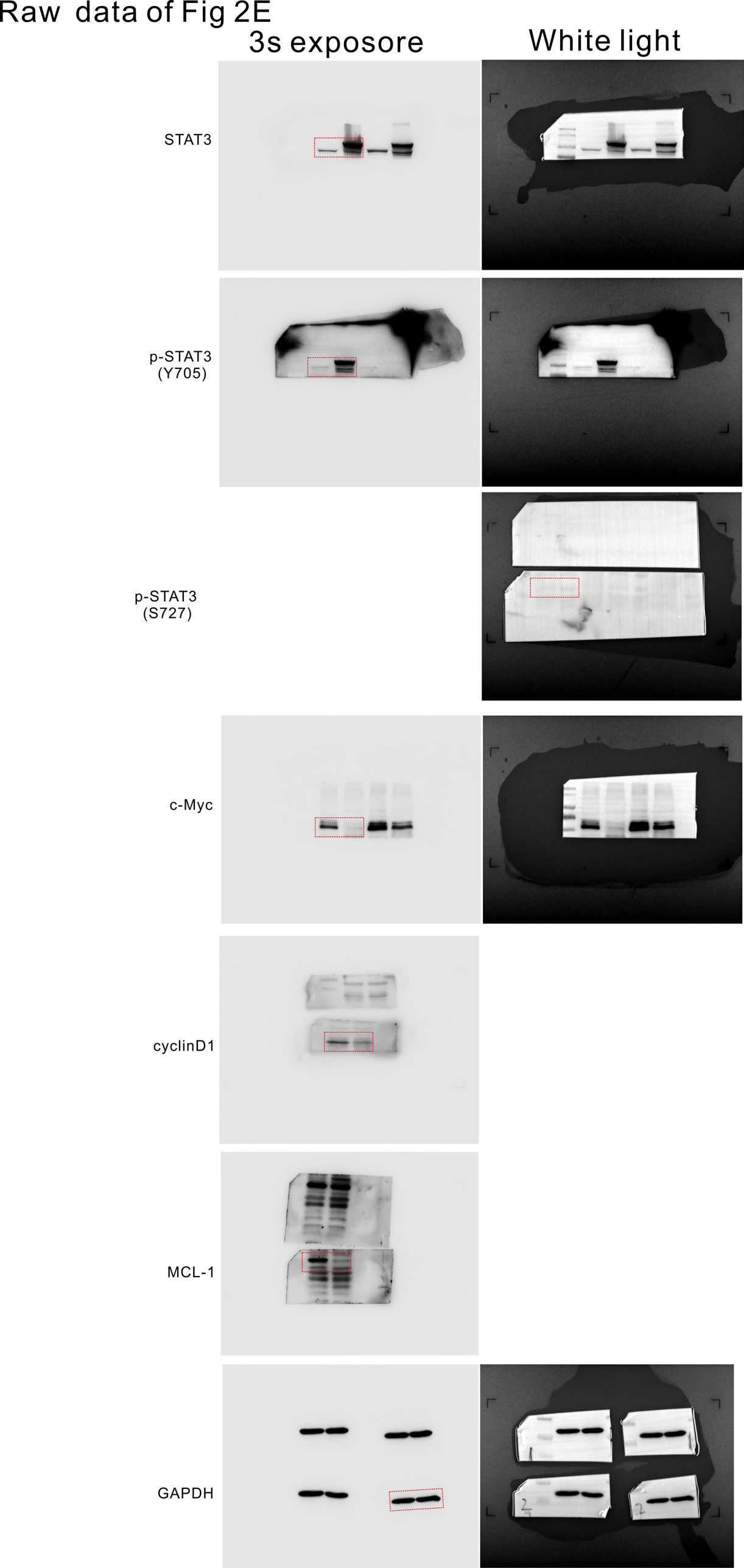

Supplement: Supplementary file 2 — Additional file 2. [file 12885_2022_9837_MOESM2_ESM.jpg]
